# Prevalence and risk factors of CKD-associated osteoporosis in maintenance hemodialysis patients aged over 50 years: a cross-sectional study

**DOI:** 10.1038/s41598-026-35136-x

**Published:** 2026-01-09

**Authors:** Yafei Bai, Yanyu Lin, Na An, Chunli Wang, Yajun Deng, Ruman Chen

**Affiliations:** 1 Blood Purification Center of Hainan General Hospital, Hainan Affiliated Hospital of Hainan Medical University, No. 19 Xiuhua Road, Haikou, 570311 Hainan China; 2 Department of Orthopedics, Xi’an Daxing Hospital, No. 4999 Shanglin Road, Xi’an, 710000 Shanxi China

**Keywords:** Chronic kidney disease, Maintenance hemodialysis, CKD-associated osteoporosis, Risk factors, Diseases, Health care, Medical research, Nephrology, Risk factors

## Abstract

CKD-associated osteoporosis is a critical complication in Chronic Kidney Disease stage 5 on Dialysis(CKD G5D) patients, increasing fracture risk and mortality. However, its prevalence and risk factors in older adults undergoing CKD G5D are still poorly understood. This study aimed to investigate the prevalence and independent risk factors of CKD-associated osteoporosis in CKD G5D patients aged over 50 years in Hainan General hospital to guide targeted prevention and management strategies. This cross-sectional study enrolled 258 CKD G5D patients from Hainan General hospital (May 2023–October 2024). Bone mineral density at the lumbar spine, femoral neck, and total hip was measured using dual-energy X-ray absorptiometry. CKD-associated osteoporosis was defined as a T-score ≤ − 2.5 or the presence of a fragility fracture. Demographic data, body measurements, and laboratory results were collected and analyzed. Variable selection was performed using a combination of LASSO regression and univariate logistic regression, followed by multivariate logistic regression to identify independent risk factors. Among the participants, 109 (42.3%) were diagnosed with CKD-associated osteoporosis. Multivariable logistic regression analysis identified female (OR = 3.44, 95%CI: 1.77–6.80, *P* < 0.01), decreased grip strength (per 1 kg decrease) (OR = 1.05, 95%CI: 1.00-1.09, *P* < 0.05), and decreased albumin levels (per 1 g/L decrease) (OR = 1.11, 95%CI: 1.01–1.22, *P* = 0.03) as independent risk factors for CKD-associated osteoporosis. CKD-associated osteoporosis is highly prevalent in CKD G5D patients over 50 years old, especially in females with low grip strength and low albumin levels. This highlights the need for enhanced screening of high-risk groups and integrating muscle function training and nutritional interventions into CKD-associated osteoporosis management.

**Supplementary Information:**

The online version contains supplementary material available at 10.1038/s41598-026-35136-x.

## Introduction

Chronic kidney disease (CKD) has become a major global public health issue, with escalating worldwide incidence and prevalence. For kidney failure, hemodialysis remains the principal treatment modality^1,2^. However, patients receiving maintenance dialysis therapy frequently develop multiple metabolic disturbances and complications^3^. Among these, CKD-associated osteoporosis significantly threatens patients’ quality of life and long-term prognosis due to its high prevalence and fracture risk. Research indicates that the incidence of CKD-associated osteoporosis in CKD G5D patients is significantly higher than in the general population^4,5^. The mortality rate in CKD G5D patients with fractures (51.1%) is significantly higher than that in the non-fracture group (37.0%)^6^. Chronic kidney disease–mineral and bone disorder (CKD-MBD) is the core pathological mechanism underlying bone metabolic disorders in dialysis patients. Factors including aging, calcium and phosphorus metabolic imbalance, vitamin D deficiency, secondary hyperparathyroidism (SHPT), and inflammation may further exacerbate CKD-associated osteoporosis progression^7^.

CKD-associated osteoporosis increases patient suffering and medical burden, and may also negatively impact long-term dialysis treatment adherence and outcomes^8^. Thus, investigating the prevalence and risk factors of CKD-associated osteoporosis in CKD G5D patients is crucial for early diagnosis, prevention, and treatment of this condition. This study reports the prevalence and risk factors of CKD-associated osteoporosis in CKD G5D patients aged over 50 years. Using a cross-sectional design, it comprehensively analyzed the current status and potential risk factors of CKD-associated osteoporosis in this population, aiming to provide a scientific basis for clinical prevention and treatment.

## Methods

### Study design and participants

**Fig. 1:**
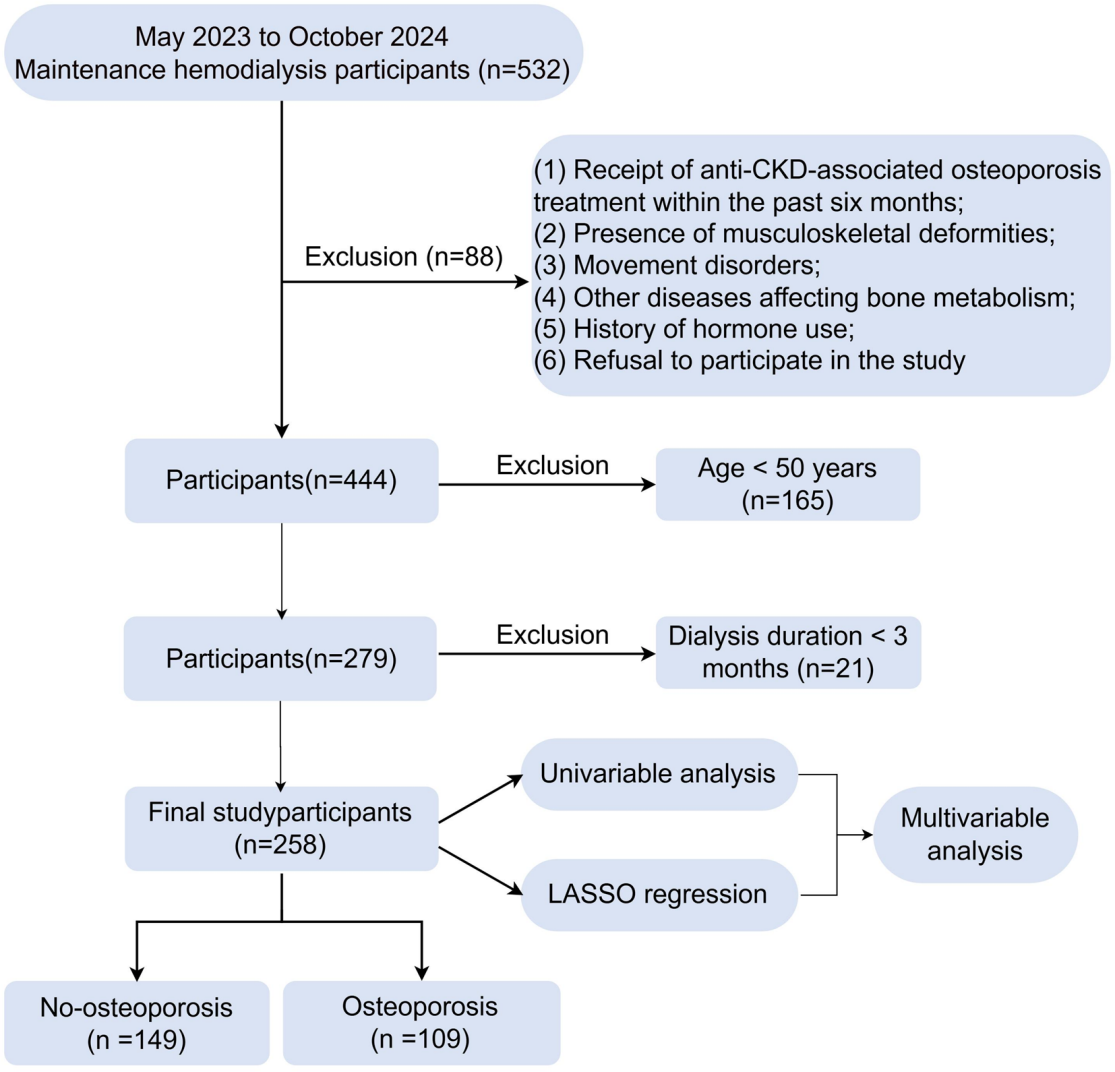
Flowchart of this study.

This cross-sectional study included 258 patients undergoing CKD G5D treatment at the Blood Purification Center of the Hainan General hospital from May 2023 to October 2024. The inclusion criteria were: (1) prospective completion of a DXA examination specifically for this study; (2) age over 50 years; (3) undergoing hemodialysis for at least three months (two to three times per week). All eligible patients undergoing maintenance hemodialysis at our center during the study period were systematically invited to undergo this DXA assessment.The exclusion criteria were: (1) inability to undergo DXA for any reason; (2) receipt of anti-osteoporosis treatment (*n* = 8,e.g., Denosumab) within the past six months; (3) presence of musculoskeletal deformities; (4) movement disorders; (5) other diseases affecting bone metabolism(Hyperthyroidism, osteomalacia: Patients with clinically suspected osteomalacia were also excluded. Suspicion was based on a combination of the following: presence of persistent bone pain and proximal muscle weakness, along with biochemical evidence of severe vitamin D deficiency (serum 25-hydroxyvitamin D level < 10 ng/mL) and/or persistently low serum calcium and phosphate levels despite adequate supplementation. While bone histomorphometry is the diagnostic gold standard, it was not performed due to its invasive nature and limited clinical availability. etc.); (6) history of hormone use; (7) refusal to participate in the study. This study was approved by the Ethics Committee of the Hainan General hospital (Ethics approval number: [2022]416) and conducted in accordance with the Helsinki Declaration. Written informed consent was obtained from all participants. The study flowchart is presented in Fig. 1.

### Collection of general data

In this study, professional medical staff collected the baseline data of participants via the electronic medical record system or a standardized questionnaire. The information included sex, age, height, weight, duration of dialysis, frequency of dialysis, type of vascular access for CKD G5D, primary kidney disease, history of osteoporotic fractures, and other underlying medical conditions.

### Laboratory biochemical indicators

To eliminate the potential bias from dialysate, blood samples were collected prior to hemodialysis in this study. Participants were required to fast overnight, with fasting venous blood collected the following morning. Laboratory tests included C-reactive protein (CRP), vitamin D, parathyroid hormone (PTH), total protein (TP), albumin (ALB), globulin (GLB), albumin/globulin (A/G) ratio, UREA, uric acid (UA), creatinine (Cr), white blood cell count (WBC), neutrophil percentage, lymphocyte percentage, hemoglobin (HGB), platelet (PLT), calcium (Ca), potassium (K), phosphorus (P), and magnesium (Mg). The eGFR was calculated using the Chronic Kidney Disease Epidemiology Collaboration (CKD-EPI) formula^9^.In this study, PTH levels were detected by electrochemiluminescence, with a normal range of 15–68.3.3 pg/ml. 25-hydroxyvitamin D was measured by chemiluminescent immunoassay, with a normal range of greater than 30 ng/ml.

### Diagnosis of CKD-associated osteoporosis

In this study, bone mineral density (BMD) of the lumbar spine (L1-L4), femoral neck, and total hip was measured using DXA (MEDILINK, France). To ensure measurement accuracy, the instrument was precisely calibrated before each test, maintaining the coefficient of variation within an acceptable range. All BMD examinations were conducted by licensed and well-trained technicians, and the BMD measurements were analyzed by a certified technical specialist. Based on the World Health Organization (WHO) criteria^10^, a T-score of ≤ − 2.5 was used as the inclusion criterion for the CKD-associated osteoporosis (OP) group, while a T-score of > − 2.5 was used for the non-CKD-associated osteoporosis (non-OP) group. Additionally, a fragility fracture in the presence of a T-score of > − 2.5 at any site was also diagnostic of CKD-associated osteoporosis.

### Measurement of grip strength and skeletal muscle mass

In this study, grip strength was measured using a WCS-10,000 dynamometer (Wanqing Electronics, Shanghai) for both dominant and non-dominant hands of participants. During measurement, participants stood with feet naturally apart and arms hanging down, gripping the dynamometer with maximum force for at least 5 s. Each hand was measured three times, with intervals of at least 60 s between measurements to allow for adequate recovery and minimize fatigue-related errors. The highest value from the three measurements was recorded as the maximum grip strength. Appendicular muscle mass was measured using a DXA (MEDILINK, France). The Skeletal Muscle Index (SMI) was calculated using the formula: appendicular muscle mass (kg)/height² (m²). The measurement procedure was as follows: Subjects wore a single layer of clothing and removed their shoes and socks to ensure full contact between their palms and soles and the metal electrodes. After entering the subject’s name, sex, age, and height into the device, it automatically measured appendicular muscle mass, body mass index (BMI), and body fat percentage, and calculated the SMI based on these parameters.

### Statistical analysis

All statistical analyses were conducted using R software (version 4.3.3). Prior to formal statistical analysis, the completeness of all variables was evaluated to address missing data. Variables with a missing rate exceeding 16% were excluded from subsequent analyses, as high missing rates may introduce unmanageable bias even after imputation. For the remaining variables with a missing rate of less than 16%, multiple imputation (MI) was performed to supplement missing values using the mice package in R software. Descriptive statistics are presented as Mean ± SD for normally distributed data and as Median with interquartile range (IQR) for non-normally distributed data. The Shapiro-Wilk test was used to assess data normality. For normally distributed variables, intergroup comparisons were performed using the independent samples t-test; for non-normally distributed variables, the Mann-Whitney U test was applied. Categorical variables are reported as frequencies and percentages (n,%) and were compared between groups using the chi-square test.

This study employed a variable screening approach combining Least Absolute Shrinkage and Selection Operator Regression (LASSO) regression and univariate logistic regression. LASSO regression, leveraging its L1 regularization, effectively identifies key variables and mitigates overfitting, particularly in the presence of multicollinearity. Variables associated with CKD-associated osteoporosis were screened using both LASSO regression and univariate logistic regression, with the intersection of their results selected for subsequent multivariate logistic regression analysis. P-values < 0.05 were considered statistically significant.

The linearity assumption of continuous variables selected for the multivariate logistic regression model was verified using the Box-Tidwell test—a standard method for validating linearity in logistic regression. For each continuous variable, a univariate logistic regression model was first constructed with CKD-associated osteoporosis status as the dependent variable. Subsequently, an interaction term between the continuous variable and logit(P) (where P denotes the probability of CKD-associated osteoporosis) was incorporated into the model. A P-value > 0.05 for this interaction term was considered indicative of a linear association between the continuous variable and logit(P), confirming compliance with the linearity assumption of the logistic regression model. All Box-Tidwell test analyses were conducted using the car package in R software.

The calibration of the multivariate logistic regression model was evaluated using the Hosmer-Lemeshow test, implemented via the ResourceSelection package in R software. Participants were stratified into 10 equal deciles according to the model’s predicted probability of CKD-associated osteoporosis. The test uses a chi-square statistic to compare the observed number of cases (actual CKD-associated osteoporosis status) and expected number of cases (model-predicted) in each decile; a P-value > 0.05 indicates good model calibration, with no significant discrepancy between observed and predicted outcomes.

## Results

### Baseline characteristics of participants

Variables with a missing rate exceeding 16% were excluded, while those with a missing rate < 16% underwent multiple imputation; the specific missing rate of each initially collected variable is provided in Supplementary Table 4. This study included 258 patients receiving CKD G5D, of whom 148 were male (57.40%) and 110 were female (42.60%). The median age was 60 years (IQR:55–67), and the median duration of hemodialysis was 48 months (IQR:21–89). Among the participants, 109 (42.30%) were in the CKD-associated osteoporosis group and 149 (57.70%) were in the non-CKD-associated osteoporosis group. Forty-eight patients were classified as having osteoporosis because of fractures occurring with a T - score above − 2.5. The primary fracture types included rib fractures, fractures of the distal tibia and fibula, and clavicle fractures. Compared with the non-CKD-associated osteoporosis group, the CKD-associated osteoporosis group exhibited a higher median age (62 vs. 59 years, *P* = 0.02), a higher proportion of females (66.97% vs. 24.83%, *P* < 0.01), a lower BMI (21.00 vs. 23.00 kg/m², *P* < 0.01), a lower SMI (4.40 ± 0.93 vs. 5.08 ± 0.98 kg/m², *P* < 0.01), lower grip strength (19.10 vs. 25.30 kg, *P* < 0.01). Additionally, significant differences were observed between the two groups in TP, UREA, Ca, K, ALB, Cr, and Mg levels (Table 1).

**Table 1:** Baseline characteristics of the study participants grouped by CKD-associated osteoporosis status.

Variables	Total(*n* = 258)	No-CKD-associated osteoporosis(*n* = 149)	CKD-associated osteoporosis(*n* = 109)	*P*-value
Age (years)	60 (55–67)	59(54–66)	62 (56–68)	0.02
sex				< 0.01
Male	148 (57.4%)	112 (75.2%)	36(33.0%)	
Female	110(42.6%)	37(24.8%)	73 (67.0%)	
BMI(kg/m^2^)	22.00 (20.00–24.17)	23.00 (21.00–25.00)	21.00 (19.14–23.01)	< 0.01
Primary kidney disease				0.53
Chronic nephritis	120(46.5%)	64(43.0%)	56(51.4%)	
Diabetic nephropathy	71 (27.5%)	42(28.2%)	29 (26.6%)	
Hypertensive nephropathy	21 (8.1%)	13 (8.7%)	8 (7.3%)	
Other	46 (17.8%)	30 (20.1%)	16 (14.7%)	
Dialysis duration (months)	48 (21–89)	38(19–88)	53(24–89)	0.16
Dialysis access				0.11
Tunneled Cuffed Catheter	38 (14.7%)	16 (10.7%)	22 (20.2%)	
Arteriovenous Graft	10(3.9%)	6(4.0%)	4 (3.7%)	
Arteriovenous Fistula	210(81.4%)	127 (85.2%)	83 (76.2%)	
Skeletal Muscle Index(kg/m^2^)	4.79 ± 1.01	5.08 ± 0.98	4.40 ± 0.93	< 0.01
Grip strength(kg)	22.95 (17.80–29.10)	25.30 (20.40–32.00)	19.10 (14.60–24.40)	< 0.01
Total Protein(g/L)	70.01 ± 5.94	70.64 ± 5.97	69.14 ± 5.81	0.04
Albumin(g/L)	38.33 (36.20–40.50)	38.90 (36.60–41.50)	37.60 (35.60–39.70)	< 0.01
Globulin(g/L)	31.86 ± 5.00	31.90 ± 5.00	31.80 ± 5.02	0.88
Albumin/Globulin ratio	1.22 (1.07–1.37)	1.22 (1.10–1.37)	1.21 (1.04–1.37)	0.29
vitaminD(ng/ml)	32.55 (26.20–39.60)	32.30 (25.00–39.10)	32.90 (27.20–39.70)	0.36
ParathyroidHormone(pg/ml)	362.23 (160.30–604.60)	380.70 (160.30–592.40)	340.00 (167.40–709.17)	0.85
Calcium(mmol/L)	2.21 ± 0.18	2.19 ± 0.18	2.24 ± 0.18	0.03
Phosphorus(mmol/L)	1.75 (1.41–2.08)	1.78 (1.41–2.10)	1.68 (1.38–2.06)	0.18
Magnesium(mmol/L)	1.06 (0.96–1.17)	1.09 (0.98–1.19)	1.04 (0.92–1.14)	0.01
Potassium(mmol/L)	4.48 ± 0.62	4.57 ± 0.57	4.37 ± 0.67	0.01
White Blood Cell(10^9^/L)	6.15 (5.20–7.43)	6.14 (5.20–7.28)	6.16 (5.22–7.54)	0.92
Neutrophil(%)	66.78 ± 8.04	66.15 ± 7.98	67.65 ± 8.09	0.14
Lymphocyte(%)	19.14 (15.00–23.48)	19.02 (15.38–23.30)	19.25 (14.38–23.88)	0.80
Hemoglobin(g/L)	115.00 (101.25–124.00)	114.00 (103.00–124.50)	115.00 (101.00–122.00)	0.62
Platelet(10^9^/L)	196.10 (153.00–236.00)	196.20 (153.00–235.00)	193.50 (156.00–252.00)	0.91
Urea Nitrogen(mmol/L)	21.20 ± 6.45	22.16 ± 6.41	19.89 ± 6.29	< 0.01
Uric Acid(µmol/L)	411.99 ± 127.22	423.95 ± 124.07	395.64 ± 130.21	0.08
Creatinine(µmol/L)	867.92 (689.00–1,106.00)	947.00 (748.00–1,203.75)	793.00 (598.00–969.00)	< 0.01

Normally distributed continuous variables are presented as Mean ± Standard Deviation; non-normally distributed variables are presented as Median (Interquartile Range, IQR). Categorical variables are presented as n (%).Remarks: TCC = Tunneled Cuffed Catheter, AVG = Arteriovenous Graft, AVF = Arteriovenous Fistula, SMI = Skeletal Muscle Index, TP = Total Protein, ALB = Albumin, GLB = Globulin, A/G ratio = Albumin/Globulin ratio, UREA = Urea Nitrogen, UA = Uric Acid, Cr = Creatinine, WBC = White Blood Cell, HGB = Hemoglobin, PLT = Platelet, Ca = Calcium, K = Potassium, P = Phosphorus, Mg = Magnesium, PTH = ParathyroidHormone.

### Selection of variables

**Fig. 2:**
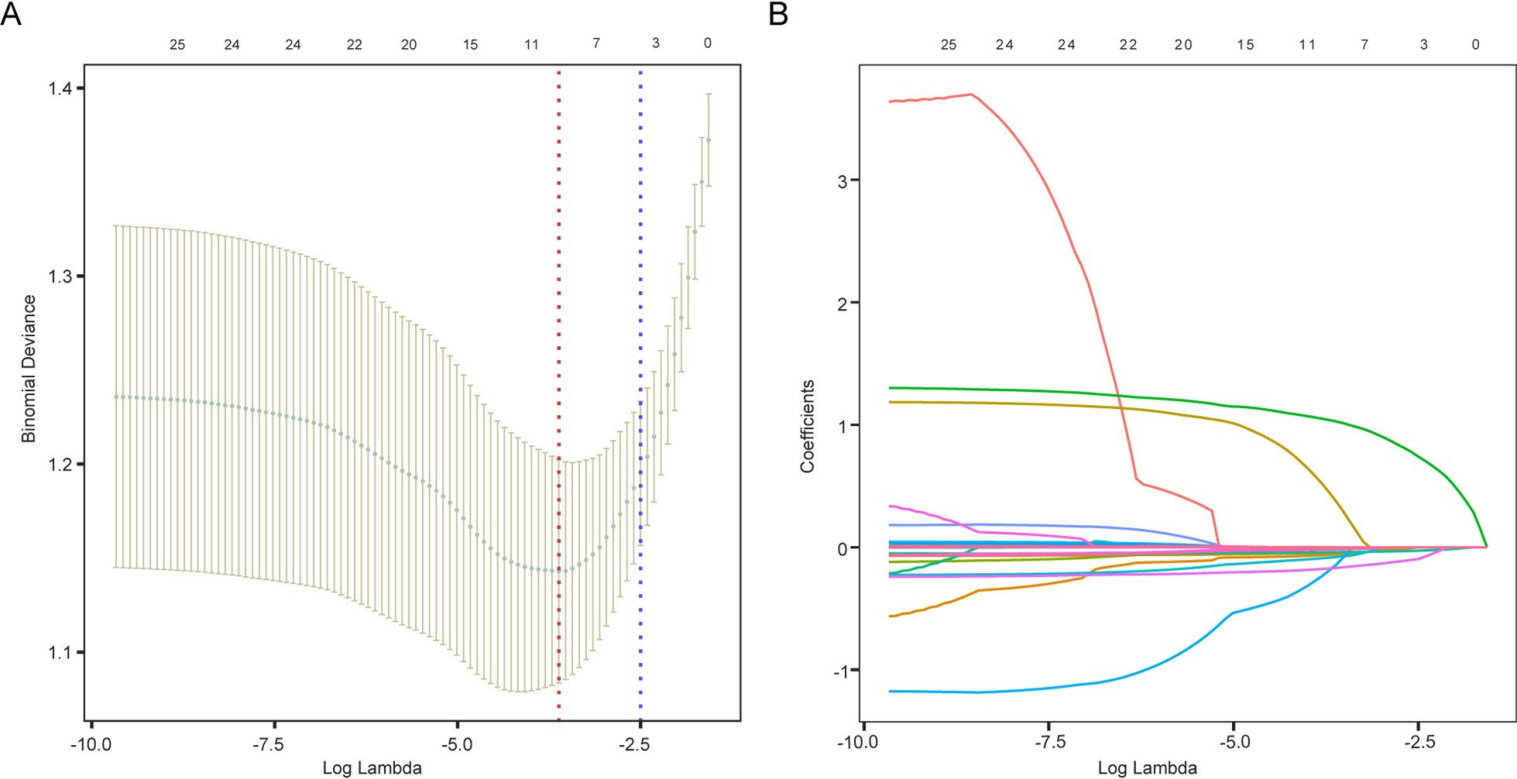
Variable selection was performed using LASSO regression. (**A**) Left dashed line represents the minimum lambda value (lambda.min), while the right dashed line marks the optimal lambda value (lambda.1se); (**B**) LASSO regression model screening variable trajectories.

Following LASSO regression with tenfold cross-validation, ten variables (sex, BMI, dialysis duration, SMI, grip strength, ALB, UREA, Ca, K, and Mg) were selected based on the lambda.min criterion (Supplementary Table 1). The processes of cross-validation and variable shrinkage are depicted in Fig. 2A and B, respectively. Univariable logistic regression analysis revealed that female, older age, lower BMI, AVF as the dialysis access, lower SMI, lower grip strength, TP, ALB, UREA, Cr, Ca, K, and Mg were all significantly associated with the risk of CKD-associated osteoporosis (Table 2). To enhance the reliability and scientific rigor of variable screening, this study integrated the results of LASSO regression and univariate logistic regression. The nine variables selected by both methods (Supplementary Fig. 1) were included in the feature set for subsequent multivariate logistic regression analysis.

**Table 2:** Univariable logistic regression analysis of potential risk factors for CKD-associated osteoporosis.

Variables	OR	95% CI	*P*-value
Sex			
Male		ref	
Female	6.14	3.59–10.71	< 0.01
Age (years)	1.03	1.01–1.06	0.02
BMI(kg/m^2^)	0.85	0.78–0.92	< 0.01
Primary kidney disease			
Chronic nephritis		ref	
Diabetic nephropathy	0.79	0.43–1.43	0.43
Hypertensive nephropathy	0.70	0.26–1.79	0.47
Other	0.61	0.30–1.22	0.17
Dialysis duration(months)	1.00	1.00–1.01.00.01	0.30
Dialysis access			
Tunneled Cuffed Catheter		ref	
Arteriovenous Graft	0.49	0.11–1.98	0.32
Arteriovenous Fistula	0.48	0.23–0.95	0.04
Skeletal Muscle Index(kg/m^2^)	0.47	0.35–0.62	< 0.01
Grip strength(kg)	0.90	0.86–0.93	< 0.01
Total Protein(g/L)	0.96	0.92–1.00.92.00	0.05
Albumin(g/L)	0.88	0.82–0.95	< 0.01
Globulin(g/L)	1.00	0.95–1.05	0.88
Albumin/Globulin ratio	0.48	0.16–1.42	0.19
vitaminD(ng/ml)	1.01	0.01–1.12	0.26
ParathyroidHormone(pg/ml)	1.00	0.00–1.08.00.08	0.28
Calcium(mmol/L)	5.00	1.21–21.68	0.03
Phosphorus(mmol/L)	0.70	0.41–1.16	0.17
Magnesium(mmol/L)	0.16	0.03–0.79	0.03
Potassium(mmol/L)	0.59	0.38–0.89	0.01
White Blood Cell(10^9^/L)	1.02	0.89–1.18	0.74
Neutrophil(%)	1.02	0.99–1.06	0.14
Lymphocyte(%)	0.99	0.96–1.03	0.79
Hemoglobin(g/L)	1.00	0.98–1.01	0.51
Platelet(10^9^/L)	1.00	1.00–1.01.00.01	0.69
Urea Nitrogen(mmol/L)	0.94	0.91–0.98	< 0.01
Uric Acid(µmol/L)	1.00	0.99–1.00.99.00	0.08
Creatinine(µmol/L)	1.00	0.99–1.00.99.00	< 0.01

Remarks: TCC = Tunneled Cuffed Catheter, AVG = Arteriovenous Graft, AVF = Arteriovenous Fistula, SMI = Skeletal Muscle Index, TP = Total Protein, ALB = Albumin, GLB = Globulin, A/G ratio = Albumin/Globulin ratio, UREA = Urea Nitrogen, UA = Uric Acid, Cr = Creatinine, WBC = White Blood Cell, HGB = Hemoglobin, PLT = Platelet, Ca = Calcium, K = Potassium, P = Phosphorus, Mg = Magnesium, PTH = ParathyroidHormone.Continuous biomarkers are modelled as OR per 1-unit decrease (grip strength, kg; albumin, g/L; SMI, kg/m²; UREA, mg/dL; Ca, mmol/L; K, mmol/L; Mg, mmol/L; vitamin D, ng/ml; PTH, pg/ml; creatinine, µmol/L; uric acid, µmol/L) or per 1-unit increase (age, years; BMI, kg/m²) as indicated. All ORs are derived from the same sample of *n* = 258 individuals.

### Verification of linearity assumption for continuous variables

To ensure the validity of the multivariate logistic regression model, the Box-Tidwell test was performed to verify the linearity of the 8 selected continuous variables. As presented in Table S3, the P-values for the “continuous variable × logit(P)” interaction terms of all variables exceeded 0.05: BMI (*P* = 0.21), SMI (*P* = 0.93), grip strength (*P* = 0.38), ALB (*P* = 0.19), UREA (*P* = 0.91), Ca (*P* = 0.76), K (*P* = 0.14), and Mg (*P* = 0.20). These results confirm that each continuous variable exhibited a linear association with the logit of the outcome variable, supporting their direct inclusion in the multivariate logistic regression model without additional variable transformation.

### Analysis of risk factors for CKD-associated osteoporosis in CKD G5D patients

**Fig. 3:**
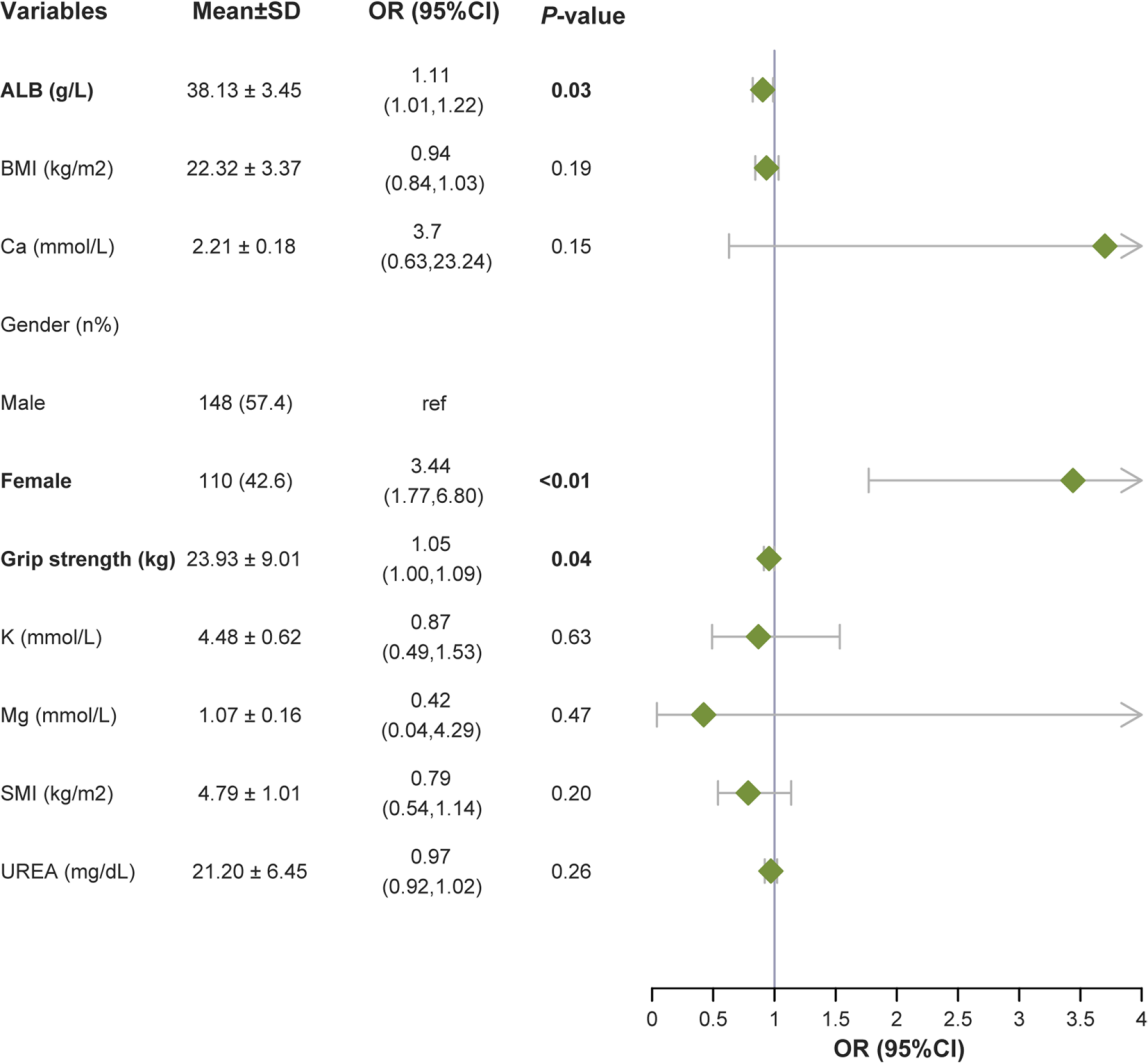
Forest plot based on multivariate logistic regression analysis. ALB = Albumin; BMI = Body Mass Index; SMI = Skeletal Muscle Index; Ca = Calcium; K = Potassium; Mg = Magnesium; UREA = Urea Nitrogen.Odds ratios for continuous variables are expressed per 1-unit decrease in the measurement, indicating the increase in odds of osteoporosis associated with each 1-unit loss. The analysis included all 258 participants.

The multivariable logistic regression analysis showed that female (OR 3.44, 95% CI 1.77–6.80), lower grip strength (per 1-kg decrease: OR 1.05, 95% CI 1.00–1.09) and lower serum albumin (per 1 g/L decrease: OR 1.11, 95% CI 1.01–1.22) were independently associated with CKD-associated osteoporosis (Fig. 3, Supplementary Table 2).

### Calibration of the multivariate logistic regression model

To verify the validity of the multivariate logistic regression model, the Hosmer-Lemeshow test was performed. As presented in Table 3, the test yielded a chi-square statistic of 2.51 (degrees of freedom = 8) and a P-value of 0.96. Since *P* > 0.05, there was no significant discrepancy between the observed number of CKD-associated osteoporosis cases and the model-predicted number across the 10 deciles of predicted probabilities. This confirms excellent model calibration—its predicted risk of CKD-associated osteoporosis is highly consistent with actual disease occurrence in the study population. Such calibration validity supports the credibility of the model’s estimates for independent risk factors.

**Table 3:** Results of the Hosmer-Lemeshow tests.

Chi-square (X²)	Degrees of Freedom	*p*-value
2.51	8	0.96

## Discussion

This study examined the prevalence and risk factors of CKD-associated osteoporosis in CKD G5D patients aged over 50 years. The results indicated that the prevalence of CKD-associated osteoporosis was 42.3%, with female, decreased grip strength, and decreased albumin levels identified as independent risk factors, with each 1 kg decrease in grip strength and each 1 g/L decrease in albumin increasing the odds of osteoporosis by 4.6% and 10.6%, respectively. These findings offer valuable insights into the epidemiological characteristics and potential mechanisms underlying CKD-associated osteoporosis in dialysis patients. Subsequent sections will compare and analyze these results with those of other relevant studies and explore possible reasons.

### Prevalence of CKD-associated osteoporosis in CKD G5D patients

The first epidemiological survey of CKD-associated osteoporosis in China revealed a prevalence of 19.2% among individuals over 50 years old. Previous studies have indicated that the incidence of CKD-associated osteoporosis in CKD G5D patients ranges from 23% to 41%^11,12^. The prevalence of CKD-associated osteoporosis in CKD G5D patients in this study was 42.3%, which is slightly higher than that reported in previous literature. This discrepancy may be attributed to the following factors. First, the proportion of females in the CKD-associated osteoporosis group in this study was 67.0%, significantly higher than the 53% reported by Hashimoto et al. in their CKD G5D cohort^12^. The decline in estrogen levels after menopause accelerates bone loss in females, consistent with previous studies showing that the risk of CKD-associated osteoporosis in females is three times higher than that in males^13^. Second, the diagnostic criteria used in this study differed from those in previous studies. The WHO criteria (T-score ≤ − 2.5) were used in this study, and some patients were diagnosed based on a history of fractures. In contrast, previous studies relied solely on the T-score of bone density, which may have led to an underestimation of the prevalence^11^. Third, geographical differences in dietary habits and sun exposure also influence the prevalence of CKD-associated osteoporosis. In Japan, the diet is rich in fish and natto products, which are high in vitamin D and calcium and are beneficial to bone health^14^. In Hainan, the diet is diverse but may lack sufficient sources of calcium and vitamin D. Although the region enjoys abundant sunshine and long daylight hours, which theoretically allows residents to synthesize adequate amounts of vitamin D through sun exposure, local residents may reduce their sun exposure time due to excessive sun protection measures, thereby affecting vitamin D synthesis. Therefore, dietary habits and sun exposure in both Japan and Hainan can influence the prevalence of CKD-associated osteoporosis.

### Sex and CKD-associated osteoporosis in CKD G5D patients

This study identified female as an independent risk factor for CKD-associated osteoporosis, consistent with previous research^15^. In this study, the proportion of females in the CKD-associated osteoporosis group was significantly higher than that in the non-CKD-associated osteoporosis group (67.0% vs. 24.8%), indicating that female CKD G5D patients should be prioritized for CKD-associated osteoporosis screening.

The elevated risk of CKD - associated osteoporosis in females with CKD G5D may be linked to several pathophysiological mechanisms. Firstly, the amplified impact of estrogen deficiency is a key factor. Postmenopausal estrogen levels decline sharply, directly increasing osteoclast activity and osteoblast apoptosis, thereby accelerating bone resorption and loss^16,17^. Secondly, sex disparities in CKD-MBD play a significant role. Postmenopausal females experience reduced cutaneous vitamin D synthesis^18,19^. Combined with the inhibition of 1α-hydroxylase activity in uremia, this results in a severe deficiency of active vitamin D^20,21^, which exacerbates CKD-MBD and secondary hyperparathyroidism (SHPT), further worsening bone loss^22,23^. Despite the lower prevalence of CKD-associated osteoporosis in males, they may still be at risk due to decreased androgen levels^24^.

Furthermore, beyond the physiological changes of menopause, CKD itself imposes an additional burden on gonadal hormone production, potentially amplifying the risk of osteoporosis. In women with CKD, there is a high prevalence of premature ovarian insufficiency and early menopause, which extends the duration of estrogen deficiency^25^. In men, CKD is a well-established cause of hypogonadism, characterized by low testosterone levels^26^. This deficiency of androgens in men contributes directly to reduced bone formation and increased resorption, a risk factor that is often underrecognized in this population. Therefore, the heightened risk of osteoporosis in CKD G5D patients is not merely additive (general population risk + CKD risk) but synergistic, driven by the compounding effects of uremia on both the skeletal and reproductive endocrine systems.

While our study was not statistically powered to conduct fully stratified multivariable analyses by sex, we have reflected on our findings in this context. The strong association between decreased grip strength and osteoporosis in the overall cohort, despite the profound difference in sex composition between groups, suggests that muscular weakness may be a universal risk factor relevant to both men and women. In contrast, the association with hypoalbuminemia was weaker in the univariate comparison and might be more susceptible to confounding by sex. It is therefore plausible to speculate that the contribution of hypoalbuminemia could be more prominent in male patients, in whom the overwhelming effect of postmenopausal estrogen deficiency is absent, allowing factors like chronic inflammation and malnutrition to play a more detectable role^27,28^. This hypothesis, generated from our data, warrants explicit testing in future studies with larger sample sizes designed for sex-stratified analysis.

### Grip strength and CKD-associated osteoporosis in CKD G5D patients

This study identified low grip strength as an independent risk factor for CKD-associated osteoporosis, consistent with recent research findings^29,30^. A cross-sectional study, including 594 middle-aged and older adults, further supports this relationship^31^. The close interaction between muscle strength and bone health, often termed the “muscle-bone unit,” can be explained through several interconnected mechanisms, which we have organized into two primary pathways:

Direct Biomechanical and Endocrine Effects of Muscle on Bone.

Skeletal muscle exerts a direct anabolic effect on bone through mechanical loading. Muscle contractions during physical activity generate forces that are a primary stimulus for bone formation and remodeling, helping to maintain bone mass and strength^32^. Beyond mechanics, muscle functions as an endocrine organ, secreting myokines that directly influence bone metabolism. For instance, Irisin has been shown to promote osteoblast differentiation and bone formation, while Myostatin inhibition can lead to increased bone mass^33^. In the context of CKD, uremic sarcopenia and muscle weakness may thus directly compromise bone health by diminishing both the mechanical stimuli and the beneficial myokine signals essential for bone maintenance.

Grip Strength as a Marker of Shared Pathophysiological Pathways.

Alternatively, decreased grip strength can serve as a surrogate for a range of common underlying pathophysiological states that concurrently detrimentally affect both muscle and bone^34,35^. These shared pathways, highly prevalent in CKD G5D patients, include:

Physical Inactivity: Disuse from sedentary behavior or comorbidities leads to simultaneous muscle atrophy and bone loss.

Malnutrition and Protein-Energy Wasting (PEW): Inadequate intake of protein, calories, and essential nutrients like vitamin D and calcium impairs the synthesis and repair of both muscle and bone tissue^27,36^.

Chronic Inflammation and Oxidative Stress: A pro-inflammatory state, characterized by elevated cytokines such as IL-6 and TNF-α, promotes muscle protein breakdown and activates osteoclast-mediated bone resorption via the RANKL/OPG pathway^28^.

Therefore, in clinical practice, assessing handgrip strength provides a simple, non-invasive means to identify patients who are likely suffering from this constellation of shared risk factors and are consequently at high risk for both sarcopenia and osteoporosis.

Thus, grip strength serves as a simple, non-invasive indicator for assessing the risk of CKD-associated osteoporosis. Regular measurement enables early identification of high-risk individuals, facilitating targeted interventions to mitigate the risk of associated diseases^31,37^.

### Albumin and CKD-associated osteoporosis in CKD G5D patients

This study demonstrated that low albumin levels were an independent risk factor for CKD-associated osteoporosis. Low albumin levels are frequently associated with elevated inflammatory markers and malnutrition, conditions commonly observed in CKD G5D patients^27^. Chronic inflammation can accelerate bone loss by activating the RANKL/OPG pathway and promoting osteoclast activity^28^. This study did not directly measure inflammatory markers. However, the median CRP level was slightly higher in the CKD-associated osteoporosis group than in the non-CKD-associated osteoporosis group. This suggests that inflammation may be involved in the progression of CKD-associated osteoporosis. Further studies are needed to verify this hypothesis.

Moreover, low albumin levels are often associated with reduced vitamin D binding protein levels, resulting in decreased levels of active vitamin D^38^. This study did not directly measure vitamin D metabolites (e.g., 1,25(OH)₂D₃), potentially omitting their key regulatory effects on bone metabolism. Malnutrition can lead to insufficient absorption of vitamin D and minerals, thereby exacerbating bone metabolic disorders. Vitamin D deficiency impairs calcium absorption, thereby increasing the risk of CKD-associated osteoporosis^39^. Malnutrition can also induce sarcopenia, which further compromises bone health^36^. Thus, optimizing nutritional status and mitigating inflammation in CKD G5D patients may be crucial strategies for preventing CKD-associated osteoporosis.

### Differences from traditional risk factors

Traditional views posit that CKD-MBD-related indicators, such as hyperphosphatemia, hypocalcemia, and PTH, are the core driving factors of bone metabolic disorders in CKD G5D patients^40^. However, in this study, phosphorus was not statistically significant in the univariate regression analysis and was not included in the final multivariate model. Calcium was included in the multivariate model analysis but was not statistically significant. This may be attributed to the following factors: Multicollinearity interference exists due to the complex feedback regulatory relationships among calcium, phosphorus, and albumin. Despite LASSO regression’s ability to mitigate multicollinearity, the independent effect of a single variable may still be attenuated. This suggests that residual kidney function may partially compensate for mineral metabolism disorders, thereby weakening the direct association between traditional indicators and CKD-associated osteoporosis^41^.

### Regarding phosphate levels and bone mineral density

An interesting finding of our study was the lack of a statistically significant association between serum phosphate levels and CKD-associated osteoporosis (*P* = 0.17), which appears to contradict well-established evidence from large population-based cohorts like the Rotterdam Study and the MrOS study^42^. One would theoretically expect an even stronger association in a dialysis cohort due to pronounced phosphate retention and a high prevalence of overt hyperphosphatemia. Several factors may explain this discrepancy.First, our study may have been underpowered to detect a significant association due to the limited sample size and the potentially complex, non-linear relationship between phosphate and bone disease. Second, and perhaps more importantly, the pathophysiology of bone disease in CKD G5D is vastly different from that in the general population. In advanced CKD, the bone itself becomes resistant to the action of Parathyroid Hormone (PTH), and the development of adynamic bone disease—characterized by low bone turnover—is common. In such states, the classic high-turnover bone loss driven by hyperphosphatemia and secondary hyperparathyroidism may be attenuated or absent. Furthermore, the majority of our patients were likely on phosphate-lowering therapies (e.g., phosphate binders) and active vitamin D analogs, which could have modulated the absolute phosphate levels and their biological impact on bone. Therefore, the isolated measurement of serum phosphate may not fully capture the intricate and heterogeneous bone metabolic status in this population, which is better characterized by bone turnover markers or bone biopsy.

### Limitations and future research directions

To avoid the confounding effect of medication on bone mineral density, we excluded patients who had received anti-osteoporosis therapy within the past six months. While this exclusion was methodologically necessary to assess the natural associations of risk factors with untreated bone disease, it may have introduced selection bias by preferentially excluding individuals with more severe osteoporosis who were already under treatment, including those with a prior fragility fracture. Consequently, the true prevalence of CKD-associated osteoporosis in our cohort may have been underestimated. Future longitudinal studies could recruit both treated and untreated patients, analyze them as separate strata, and report combined and stratified prevalence estimates to provide a more comprehensive epidemiological picture.

This study innovatively integrated LASSO regression and logistic regression for variable selection, which effectively reduced multicollinearity interference. However, the cross-sectional design precluded the establishment of causality. Additionally, the absence of bone turnover markers or radiological fracture data may limit the analysis of CKD-associated osteoporosis subtypes. Future studies may employ longitudinal cohort designs to further elucidate the pathogenesis of CKD-associated osteoporosis and its relationship with muscle-bone interactions. Additionally, the study sample was confined to Hainan Province, which may introduce geographical selection bias. Future studies could expand the sample size and cover more regions to enhance the generalizability of the results. Finally, we excluded patients with clinically suspected osteomalacia to reduce etiological heterogeneity. Although this sharpens the focus on the osteoporosis phenotype, it may limit the generalizability of our findings to the broader CKD G5D population, in whom varying degrees of mineralization defects can coexist with low bone volume. Future studies incorporating bone turnover markers (e.g., bone-specific alkaline phosphatase, P1NP, CTX) could help to non-invasively distinguish between different types of renal osteodystrophy and better characterize the study population. Future research could further elucidate these mechanisms by incorporating techniques such as bone biopsy.

## Conclusion

In summary, this study highlights the high prevalence of CKD-associated osteoporosis in CKD G5D patients over 50 years old, with female sex, decreased grip strength, and low albumin levels identified as independent risk factors. These findings underscore the need for enhanced screening of high-risk groups and the integration of muscle function training and nutritional interventions into the comprehensive management of CKD-associated osteoporosis. Future research should focus on elucidating the pathogenesis of CKD-associated osteoporosis and developing targeted preventive and therapeutic strategies to improve the quality of life and long-term prognosis of CKD G5D patients.

## Supplementary Information

Below is the link to the electronic supplementary material.

Supplementary Material 1

Supplementary Material 2

Supplementary Material 3

Supplementary Material 4

Supplementary Material 5

## Data Availability

The datasets generated during and/or analysed during the current study are available from the corresponding author on reasonable request.
