# Supplementary material for: Prevalence and risk factors of CKD-associated osteoporosis in maintenance hemodialysis patients aged over 50 years: a cross-sectional study
Source: Sci Rep. 2026 Jan 9;16:4908. doi: 10.1038/s41598-026-35136-x (PMC12873348; doi:10.1038/s41598-026-35136-x)
Supplement: Supplementary file 1 — Supplementary Material 1 [file 41598_2026_35136_MOESM1_ESM.docx]

Table S1 LASSO regression analysis

| Variables | coeff_lamda |
| --- | --- |
| Gender | 1.0283 |
| Age (years) | 0 |
| BMI(kg/m^2^) | -0.0461 |
| Primary renal disease | 0 |
| Dialysis duration(months) | 0.0001 |
| Dialysis access | 0 |
| SMI(kg/m^2^) | -0.1691 |
| Grip strength(kg) | -0.0398 |
| TP(g/L) | 0 |
| ALB(g/L) | -0.0577 |
| GLB(g/L) | 0 |
| A/G ratio | 0 |
| UREA(mg/dL) | -0.0171 |
| UA(μmol/L) | 0 |
| Cr(μmol/L) | 0 |
| eGFR(mL/min/1.73m²) | 0 |
| WBC(10^9^/L) | 0 |
| Neutrophil(%) | 0 |
| Lymphocyte(%) | 0 |
| HGB(g/L) | 0 |
| PLT(10^9^/L) | 0 |
| Ca(mmol/L) | 0.3790 |
| K(mmol/L) | -0.0795 |
| P(mmol/L) | 0 |
| Mg(mmol/L) | -0.1448 |

Remarks: BMI = Body Mass Index; SMI = Skeletal Muscle Index; TP = Total Protein; ALB = Albumin; GLB = Globulin; UREA = Urea Nitrogen; UA = Uric Acid; Cr = Creatinine; eGFR = Estimated Glomerular Filtration Rate; WBC = White Blood Cell Count; HGB = Hemoglobin; PLT = Platelet Count; Ca = Calcium; K = Potassium; P = Phosphorus; Mg = Magnesium
