# Supplementary material for: Prevalence and risk factors of CKD-associated osteoporosis in maintenance hemodialysis patients aged over 50 years: a cross-sectional study
Source: Sci Rep. 2026 Jan 9;16:4908. doi: 10.1038/s41598-026-35136-x (PMC12873348; doi:10.1038/s41598-026-35136-x)
Supplement: Supplementary file 2 — Supplementary Material 2 [file 41598_2026_35136_MOESM2_ESM.docx]

Table S2 Multivariable logistic regression analysis for osteoporosis in MHD patients

| Variables | OR | 95% CI | *P*-value |
| --- | --- | --- | --- |
| Gender |  |  |  |
| Male |  | ref |  |
| Female | 3.4394 | 1.7701-6.8022 | 0.0003 |
| BMI(kg/m^2^) | 0.9349 | 0.8441-1.0342 | 0.1873 |
| SMI(kg/m^2^) | 0.7857 | 0.5392-1.1375 | 0.2018 |
| Grip strength(kg) | 1.0459 | 1.0015-1.0945 | 0.0471 |
| ALB(g/L) | 1.1055 | 1.0098-1.2147 | 0.0324 |
| UREA(mg/dL) | 0.9707 | 0.9211-1.0218 | 0.2586 |
| Ca(mmol/L) | 3.6996 | 0.6290-23.2413 | 0.1531 |
| K(mmol/L) | 0.8686 | 0.4906-1.5339 | 0.6267 |
| Mg(mmol/L) | 0.4219 | 0.0401-4.2922 | 0.4677 |

Remarks: ALB = Albumin; BMI = Body Mass Index; SMI = Skeletal Muscle Index; Ca = Calcium; K = Potassium; Mg = Magnesium; UREA = Urea Nitrogen
