# Supplementary material for: Prevalence and risk factors of CKD-associated osteoporosis in maintenance hemodialysis patients aged over 50 years: a cross-sectional study
Source: Sci Rep. 2026 Jan 9;16:4908. doi: 10.1038/s41598-026-35136-x (PMC12873348; doi:10.1038/s41598-026-35136-x)
Supplement: Supplementary file 3 — Supplementary Material 3 [file 41598_2026_35136_MOESM3_ESM.docx]

| Variables | Estimate | P-value |
| --- | --- | --- |
| BMI:logit_p | -0.0725 | 0.2146 |
| SMI:logit_p | -0.0139 | 0.9298 |
| grip_strength:logit_p | -0.0126 | 0.3831 |
| ALB:logit_p | 0.0865 | 0.1911 |
| UREA:logit_p | -0.0047 | 0.9075 |
| Ca:logit_p | -0.5366 | 0.7568 |
| K:logit_p | -0.9361 | 0.1376 |
| Mg:logit_p | -2.2278 | 0.1971 |

**Table S3** Results of Box-Tidwell Test for Continuous Variables
