# Supplementary material for: Prevalence and risk factors of CKD-associated osteoporosis in maintenance hemodialysis patients aged over 50 years: a cross-sectional study
Source: Sci Rep. 2026 Jan 9;16:4908. doi: 10.1038/s41598-026-35136-x (PMC12873348; doi:10.1038/s41598-026-35136-x)
Supplement: Supplementary file 4 — Supplementary Material 4 [file 41598_2026_35136_MOESM4_ESM.docx]

| **VariableNames** | **level** | **Overall** | **Missing (%)** |
| --- | --- | --- | --- |
| n |  | 258 |  |
| Age(years) |  | 62.12±8.93 | 0 |
| BMI(kg/m2) |  | 22.4±3.37 | 5 |
| Dialysis duration (months) |  | 63.57±57.11 | 0 |
| SMI(kg/m2) |  | 4.84±1.02 | 8.5 |
| Grip strength(kg) |  | 24.04±9.23 | 15.5 |
| TP(g/L) |  | 69.93±5.95 | 1.9 |
| ALB(g/L) |  | 38.14±3.44 | 1.9 |
| GLB(g/L) |  | 31.78±5.01 | 1.9 |
| A/G ratio |  | 1.23±0.23 | 1.9 |
| UREA(mg/dL) |  | 21.23±6.48 | 1.2 |
| UA(μmol/L) |  | 412.12±127.8 | 1.2 |
| Cr(μmol/L) |  | 903.9±305.85 | 1.2 |
| eGFR (mL/min/1.73m²) |  | 12.32±9.11 | 1.2 |
| WBC(10^9^/L) |  | 6.39±1.74 | 0 |
| NE_percent(%) |  | 66.78±8.04 | 0 |
| LYM_percent(%) |  | 19.8±6.32 | 0 |
| HGB(g/L) |  | 111.99±17.55 | 0 |
| PLT(10^9^/L) |  | 200.5±65.9 | 0 |
| Ca(mmol/L) |  | 2.21±0.18 | 0 |
| K(mmol/L) |  | 4.48±0.62 | 0 |
| P(mmol/L) |  | 1.78±0.49 | 0 |
| Mg(mmol/L) |  | 1.07±0.16 | 0 |
| group (%) | 0 | 149 (57.75) | 0 |
| group (%) | 1 | 109 (42.25) |  |
| gender (%) | 0 | 148 (57.36) | 0 |
| gender (%) | 1 | 110 (42.64) |  |
| primary_renal_disease (%) | 0 | 120 (46.69) | 0.4 |
| primary_renal_disease (%) | 1 | 70 (27.24) |  |
| primary_renal_disease (%) | 2 | 21 (8.17) |  |
| primary_renal_disease (%) | 3 | 46 (17.90) |  |
| dialysis_access (%) | 0 | 38 (14.73) | 0 |
| dialysis_access (%) | 1 | 10 (3.88) |  |
| dialysis_access (%) | 2 | 210 (81.40) |  |

**Table S4** Missing rate of variable

group: 0 = Normal; 1 = Osteoporosis

primary_renal_disease: 0 = Chronic nephritis; 1 = Diabetic nephropathy; 2 = Hypertensive nephropathy; 3 = Others

dialysis_access: 0 = TCC; 1 = AVG; 2 = AVF

Remarks: BMI = Body Mass Index; SMI = Skeletal Muscle Index; TP = Total Protein; ALB = Albumin; GLB = Globulin; A/G ratio = Albumin/Globulin Ratio; UREA = Urea; UA = Uric Acid; Cr = Creatinine; eGFR = Estimated Glomerular Filtration Rate; WBC = White Blood Cell Count; NE_percent = Neutrophil Percentage; LYM_percent = Lymphocyte Percentage; HGB = Hemoglobin; PLT = Platelet Count; Ca = Calcium; K = Potassium; P = Phosphorus; Mg = Magnesium.
