# Supplementary material for: Prevalence and risk factors of CKD-associated osteoporosis in maintenance hemodialysis patients aged over 50 years: a cross-sectional study
Source: Sci Rep. 2026 Jan 9;16:4908. doi: 10.1038/s41598-026-35136-x (PMC12873348; doi:10.1038/s41598-026-35136-x)
Supplement: Supplementary file 5 — Supplementary Material 5 [file 41598_2026_35136_MOESM5_ESM.pdf]

Supplementary Figure 1. LASSO regression coefficient path for variable selection.

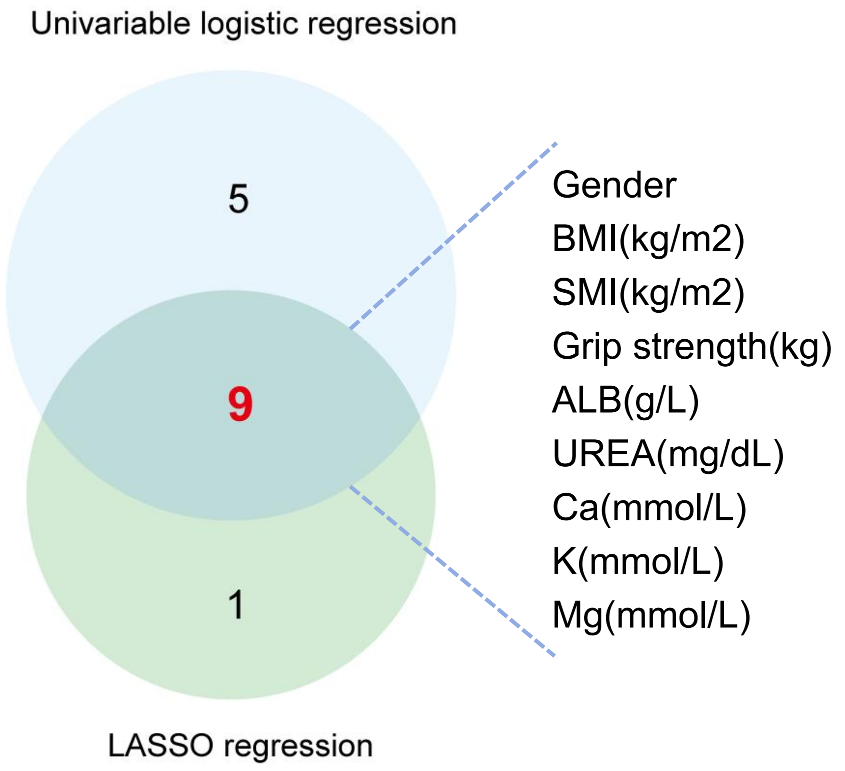

Remarks: ALB = Albumin; BMI = Body Mass Index; SMI = Skeletal Muscle Index; Ca = Calcium; K = Potassium; Mg = Magnesium; UREA = Urea Nitrogen

LASSO regression coefficient path showing how variable coefficients shrink as the penalty parameter (λ) increases. The optimal λ (vertical dashed line) was chosen by 10-fold cross-validation, retaining 9 non-zero predictors: Gender, BMI, SMI, grip strength, ALB, UREA, Ca, and K.
